# Heart Rate Variability Prediction of Stimulant-Induced Creativity Gains in Attention-Deficit/Hyperactivity Disorder

**DOI:** 10.3390/jcm14103570

**Published:** 2025-05-20

**Authors:** Carrina Appling, Nanan Nuraini, Eric Hart, David Wang, Aneesh Tosh, David Beversdorf, Bradley Ferguson

**Affiliations:** 1 Interdisciplinary Neuroscience Program, University of Missouri, Columbia, MO 65211, USA; cbaqpd@missouri.edu (C.A.); nn3w4@missouri.edu (N.N.); 2 Molecular Life Sciences Fellowship, University of Missouri, Columbia, MO 65211, USA; 3 Fulbright United States Student Program, University of Missouri, Columbia, MO 65211, USA; 4 Department of Health Psychology, University of Missouri, Columbia, MO 65211, USA; harte@health.missouri.edu; 5 Department of Emergency Medicine, University of Tennessee-Chattanooga, Chattanooga, TN 37403, USA; davidcopewang@gmail.com; 6 Department of Pediatrics, University of Missouri, Columbia, MO 65211, USA; tosha@health.missouri.edu; 7 Departments of Radiology, Neurology & Psychological Sciences, University of Missouri, Columbia, MO 65211, USA; 8 Department of Neurology, School of Medicine, University of Missouri, Columbia, MO 65211, USA; 9 Thompson Center for Autism & Neurodevelopment, University of Missouri, Columbia, MO 65211, USA

**Keywords:** stimulant medication, attention-deficit hyperactivity disorder, creativity, dopamine, norepinephrine, autonomic nervous system, heart rate variability

## Abstract

**Background/Objectives:** Attention-deficit/hyperactivity disorder (ADHD) is a highly prevalent condition etiologically related to suboptimal levels of dopamine (DA) and norepinephrine (NE) that is typically treated with psychostimulant medication. In individuals with ADHD, divergent thinking abilities have been shown to improve with the use of psychostimulants. Furthermore, psychostimulants affect autonomic nervous system (ANS) functioning, which can impact creative cognition. However, it is not known how DA and NE affect creative cognition in this setting and how this effect is related to autonomic activity in ADHD. Therefore, our objective was to elucidate ANS function and its relationship with divergent creativity gains related to psychostimulant treatment in ADHD. **Method:** Seventeen individuals diagnosed with ADHD (age 27.9 ± 6.7 sd) participated in two counterbalanced sessions—one while on their prescribed stimulant medication and another after abstaining for at least 24 h. During each session, participants completed convergent (anagrams) and divergent (Torrance Test of Creative Thinking) thinking tasks. An 8 min electrocardiogram prior to cognitive testing was taken to measure heart rate variability (HRV), which is an index of ANS functioning. **Results:** The hypothesized baseline pNN50 HRV measure was not predictive of enhanced creativity gains on convergent anagrams or divergent creativity on the Torrance when taking stimulants. **Conclusions:** In this pilot study, the relationship between baseline HRV and the impact of stimulants on anagram performance suggests the noradrenergic system may not play a role in the effect of stimulants on convergent or divergent creativity. The lack of a relationship between baseline HRV and stimulant-related changes in TTCT and anagram scores lends some support to the hypothesis that dopaminergic effects may be the predominant factor in the effect of stimulants on creativity in ADHD. Future research should further investigate the interaction between hypoactive neurotransmitter systems, particularly dopamine in divergent and norepinephrine in convergent creativity, using neuroimaging techniques to assess neurotransmitter dynamics during creativity-based tasks.

## 1. Introduction

Attention-deficit/hyperactivity disorder (ADHD) etiology is believed to be largely influenced by genetic factors, with alterations in norepinephrine (NE) and dopamine (DA) levels observed. Research indicates that decreased levels of these neurotransmitters are associated with a higher prevalence of ADHD symptoms [1]. The executive control networks, which collectively govern planning, goal-directed behavior, inhibitory control, and cognitive flexibility, exhibit reduced activation, as well as weakened intrinsic connectivity in those with ADHD compared to neurotypical individuals [2]. Over 50% of individuals with ADHD receive psychostimulant pharmacological treatment as part of their intervention strategy [3]. First-line psychostimulant medications, such as methylphenidate and amphetamines alter catecholamine levels which improve focus and impulse control [4] (Mechler et al., 2022). These pharmacotherapies act as DA and NE agonists, inhibiting the reuptake of catecholamines to increase their availability in the synaptic cleft [5,6]. This mechanism elevates DA/NE neurotransmitter levels while simultaneously reducing the metabolism of monoamines [7].

DA influences the production and activity levels of NE, a key mediator in ANS functioning. DA is also fundamental to the initiation and planning of movement, the regulation of activation states, attentional shifting, adaptation to novel stimuli, and reward processing [8]. Individuals with ADHD often have relatively low DA levels which impairs neurotransmitter signaling, contributing to attention and impulse control difficulties. Overactive DA transporters may contribute to ADHD by rapidly clearing DA from the synapses, thereby reducing its availability [9]. The ventral tegmental area (VTA) is the largest producer of DA and has been implicated in many mental health conditions, including ADHD. Research has also shown that noradrenergic inputs from the locus coeruleus (LC) to the VTA can modulate neuronal activity in the VTA, and are implicated in the effects of psychostimulants [10,11]. Dysregulated DA can lead to imbalances in NE signaling, disrupting related cognitive and physiological processes [12]. NE is essential for modulating arousal, enhancing cortical signal-to-noise ratios, coordinating state-dependent cognitive processes, and preparing the brain for rapid responses to salient stimuli [9]. There is growing recognition in the literature of multiple contributing pathways to the etiology of ADHD symptoms. Executive dysfunction theory posits that hypoactive brain regions are associated with impairments in executive control. This dysfunction, often tied to prefrontal cortex (PFC) impairments, is believed to stem from lowered NE/DA signaling [13]. In contrast, state regulation theory proposes that dysregulated autonomic nervous system (ANS) functioning mediates the altered/lowered brain arousal seen in patients with ADHD [13]. This theory is backed by the systematic review conducted by Bellato et al. [14]. wherein the characteristics of individuals with ADHD were examined, identifying ANS neurotransmission dysregulation as a consequence of DA/NE hypo-functioning. The dopaminergic mesocortical pathway and the locus coeruleus have roles in the state regulation theory brought about by dopaminergic and LC-NE deficiency [15]. Both the executive dysfunction theory and the state regulation theory share a possible relationship with altered DA/NE activity. Thus, whether ADHD pathogenesis is seen as top-down control (executive dysfunction) or bottom-up regulation (state regulation), disruptions in DA/NE neurotransmission appear to be a common underlying factor contributing to the cognitive and behavioral symptoms of ADHD [16].

Reduced arousal, which is regulated by the ANS, is believed to influence ADHD-related behaviors like impaired self-control, working memory deficits, difficulties in emotional regulation, response inhibition issues, and slowed cognitive processing through underlying neurophysiological mechanisms [17,18]). The ANS comprises the parasympathetic nervous system (i.e., rest-and-digest) and the sympathetic nervous system (i.e., fight-or-flight) and regulates several physiological processes, including cardiac function, blood pressure, and heart rate. The vagus nerve plays a crucial role in mediating parasympathetic efferent activity leading to inhibitory chronotropy and reducing cardiac tone. Additionally, the vagus nerve transmits afferent information from the heart to the locus coeruleus (LC), which is the largest producer of NE in the brain and is associated with the sympathetic response. Individuals with ADHD have demonstrated reduced heart rate variability (HRV) [19], which is associated with increased arousal and decreased sustained attention [20]. HRV, as captured by an electrocardiogram (ECG), provides a reliable, non-invasive measure of ANS function. HRV reflects both the time- and frequency-domain characteristics of cardiac autonomic control. Time-domain indices, such as the percentage of successive intervals differing by more than 50 ms (pNN50), the root mean square of successive differences (RMSSD), the standard deviation of NN intervals (SDNN), and the absolute count of NN50 intervals, quantify beat-to-beat variability largely influenced by parasympathetic (vagal) tone [21]. Frequency-domain measures decompose heart rate oscillations into spectral components: high-frequency (HF) power reflects respiratory-linked parasympathetic activity; low-frequency (LF) power represents a mix of sympathetic and parasympathetic influences; and very-low-frequency (VLF) power is thought to capture longer term regulatory processes, including thermoregulation and sympathetic modulation [22]. To date, HRV metrics have not yet been examined to assess how baseline autonomic functioning might relate to stimulant-induced changes in creativity performance.

Research indicates that the noradrenergic and dopaminergic systems may have distinct roles in modulating various aspects of creativity [23]. Creativity is classically defined as having the power or ability to create, marked by originality [24]. There are two types of tasks utilized to assess performance in terms of creativity. Convergent tasks involve searching through a wide conceptual space but require the participant to converge on one correct answer as result of this effort, while divergent tasks involve a search for an unconstrained set of multiple potential ‘creative’ responses [23]. Evidence suggests that diminished noradrenergic activity may enhance performance on tasks involving constrained cognitive flexibility/creativity, such as anagram solving, indicating a potential role for the noradrenergic system in facilitating certain types of creative problem-solving [23]. Individuals with ADHD tend to achieve significantly higher creativity scores compared to those without. A study conducted by Girard-Joyal and Gauthier (2022) demonstrated that individuals diagnosed with ADHD exhibit increased levels of creativity, as indicated by both self-reported measures and performance outcomes on the Torrance Test of Creative Thinking (TTCT), which exhibited a strong correlation with symptom severity [25]. How these relationships are altered by the presence of psychostimulant medications is not well understood. NE has been found to reduce creative performance on convergent thinking tasks, while DA has been shown to exhibit an inverted U-shaped relationship with creativity, influencing divergent thinking [23]. Previously, in this lab, research found that individuals with ADHD improved markedly on divergent tasks in the presence of psychostimulant medication. Specifically, those taking psychostimulants demonstrated enhanced fluency, flexibility, and originality scores on the TTCT [26]. Sayalı et al. (2023) investigated whether creativity levels are linked to heterogeneity in baseline DA levels and found that in those with low DA, synthesis divergence was reduced, and it was enhanced in those with higher DA in the presence of methylphenidate [27]. Spontaneous eye blink rate, an indirect measure of DA, has been linked with originality and flexibility, divergent thinking categories, in a similar inverted U-shape pattern [28]. With both insufficient and excessive DA levels leading to increased ADHD symptoms and alterations in creativity, it suggests the existence of an optimal DA balance for peak functioning. Identifying and maintaining this balance may be crucial for improving patient outcomes with psychostimulant medications.

This collective research prompted an examination of the specific roles of NE and DA in the context of creative cognition in individuals with ADHD. Our current study explored the NE aspect by investigating ANS functioning, measured by HRV, and its link to creativity in ADHD in the context of psychostimulants. We hypothesized that baseline HRV time-domain pNN50 would predict heightened creativity performance on the TTCT and on anagrams in the presence of psychostimulant medications. The pNN50 was selected as our primary outcome measure as a marker of sympathetic/parasympathetic balance, and as previous work has demonstrated that it predicted the effect of a noradrenergic agent (in that case, a beta-adrenergic antagonist, propranolol) on anagram performance in an autism spectrum disorder patient population [29].

## 2. Methods

In the current study, 17 individuals diagnosed with ADHD who were prescribed and were currently taking psychostimulant medication (mean age = 27) participated in two counterbalanced sessions in the Cognitive Neuroscience Laboratory at the University of Missouri (Columbia, MO, USA) (Table 1). Participants were excluded if they had a neurodevelopmental disorder other than ADHD, such as autism spectrum disorder or a learning disability such as dyslexia. Participants were also excluded if they had other neurological conditions, such as a seizure disorder. One session was conducted within two hours of the participants taking their prescribed stimulant medication, ensuring that the drug was active in their system. The other session occurred after participants had abstained from their prescribed stimulant for a full 24 h period or longer, allowing sufficient time for the medication to be fully metabolized and cleared. During both sessions, individuals were administered a brief IQ test and a structured series of cognitive tasks designed to assess problem-solving skills and creative thinking in a randomized order. Specifically, individuals completed the North American Adult Reading Test (NAART) (a brief assessment of verbal intelligence that is correlated with verbal performance and full-scale IQ [30]; and all participants scored within the average IQ range (VIQ = 108.47 ± 6.12; PIQ = 109.85 ± 2.89; FSIQ = 110.07 ± 5.37). This was followed by an anagram assessment and the TTCT (order randomized) to evaluate their capacity for divergent thinking and their ability to produce novel ideas, providing a comprehensive evaluation of each participant’s creative and cognitive flexibility under both medicated and unmedicated conditions.

Participants completed the verbal component of the TTCT, which included tasks designed to evaluate diverse thought processes and problem-solving abilities related to divergent verbal creativity [31]. Each participant completed either version A or B of the TTCT in both testing sessions. The assessment required participants to respond to a series of six activities (activities one through five and seven), which included prompts across the following categories: ask-and-guess, understanding context, describing actions in a scene, predicting potential consequences, suggesting product improvements, identifying unconventional uses for items, and considering the implications of improbable situations.

To illustrate, in the ‘ask-and-guess’ activity from version A, participants viewed an image and were instructed to generate questions that extended beyond observable details. The total number of relevant questions generated constituted the fluency score. Originality was measured by identifying the questions absent from a predefined list of typical responses. Flexibility was scored by examining the breadth of thematic categories represented in the questions, such as inquiries related to the image and environmental context. Responses were independently rated by two evaluators, and the mean of their scores served as the final score for each dimension. For each participant, scores from the six activities were averaged to generate composite scores for fluency, originality, and flexibility under each testing condition. Responses were scored using the TTCT scoring manual across three dimensions: fluency, originality, and flexibility [31]. Fluency represented the total number of relevant responses. Originality assessed the uniqueness of responses, awarding points for answers not found on a predetermined list of common responses. Flexibility evaluated the range of cognitive categories reflected in the responses, assigning a point for each distinct conceptual category represented.

Participants also completed a timed anagram-solving task involving one of two randomized stimulus sets, each comprising 20 anagram items (14 five-letter and 6 seven-letter anagrams). A maximum of 120 s was permitted for each trial. An example item included the scrambled string “RETCHAP,” for which the correct solution was “CHAPTER”. Latency was operationalized as the time (in seconds) taken to generate a correct solution. Trials in which no correct solution was provided within the allotted timeframe were assigned a maximum latency value of 120 s. To normalize the distribution of response times and minimize the influence of extreme values, latency data were subjected to natural logarithmic transformation, consistent with established procedures in prior research using this paradigm [32].

In addition to these cognitive assessments, we examined ANS functioning at rest during the unmedicated session. At rest, participants underwent an 8 min ECG recording, which was used to assess HRV—a key physiological marker associated with ANS function, cognitive flexibility, and emotional regulation. This physiological component acquired while off medication as a baseline allowed for an exploration of potential interactions between stimulant medication, creativity, and ANS activity. Three Ag-AgCl disposable snap electrodes containing liquid electrolyte gel were placed on the participant’s body, one under each clavicle (i.e., below the left and right collarbone) and one just below the left umbilicus. The leads were connected to an ECG100C (BIOPAC Systems, Inc., Goleta, CA, USA) amplifier attached to a BIOPAC MP150 data acquisition system (BIOPAC Systems, Inc., Goleta, CA, USA) which was connected to a research-dedicated laptop computer via an ethernet cable. Participants were instructed to rest, minimize movement, and breathe normally while electrocardiogram data were collected for eight minutes, three minutes passed for acclimation, and five more for data collection, consistent with previous research in this lab. All data were obtained with the use of Acknowledge 4.1 software (BIOPAC Systems, Inc., Goleta, CA, USA). This design was used during both sessions to detect changes found in the presence or absence of stimulant medication.

Our sample included individuals who were previously diagnosed and prescribed extended-release formulations of stimulants, both generic and brand-name stimulant medications. Dextroamphetamine (n = 8, mean = 23.5, mg ± 7.09 mg) and lisdexamfetamine (n = 5, mean = 44, mg ± 10.2 mg) were the most common (Table 1).

The study was conducted in accordance with the Declaration of Helsinki, and approved by the University of Missouri Health Sciences Institutional Review Board (HSIRB Protocol number: 2007763; Date of approval: 26 June 2017).

## 3. Analysis

Using AcqKnowledge Data Acquisition and Analysis Software version 4.1, ECG R-R intervals were processed and transformed, using the aforementioned five-minute period, into a readable text file which was then imported to Kubios HRV Scientific Software version 4.1.2.1. Frequency- and time-domains were then extracted for baseline HRV analysis after the same algorithmic artifact correction was applied to all participant data, in accordance with previous research [26,33]. One participant was excluded due to extensive artifact adjustment (i.e., 76% of all heartbeats were corrected by the artifact and ectopic beat correction algorithm) for both anagrams and TTCT analysis.

Two independent evaluators assessed all TTCT submissions, and the final scores were calculated as an average of the two scorers’ results, establishing inter-rater reliability for both stimulant conditions. The on/off stimulant condition change score was calculated (i.e., on stimulant score minus off stimulant score equaled change score) using the averaged TTCT results from the two scorers (for fluency, flexibility, and originality). The evaluators were trained on the TTCT and established reliability at the beginning of the study.

The HRV data from baseline (off stimulants) and the TTCT change scores were exported to R statistical software (version 4.5.0) for further analysis. A paired two-tailed *t*-test was performed to compare baseline heart rate variability measures for the on and off stimulant medication conditions to ensure no effect was found. Following this, we employed the statistical software R to conduct multiple linear regression analyses (*p* < 0.05) of the TTCT and anagram change scores (on stim-off stim = change score) and of the baseline HRV time- and frequency-domain variables to assess significance.

## 4. Results

### 4.1. Convergent

Linear regressions were conducted to examine whether individual HRV and heart rate (HR) metrics predicted changes in anagram task performance. None of the predictors reached statistical significance. Max HR demonstrated a positive, but nonsignificant association (*β* = 0.419, *p* = 0.120, R^2^ = 0.176), followed by STD HR (*β* = 0.406, *p* = 0.133, R^2^ = 0.165). Mean RR exhibited a moderately negative but nonsignificant association (*β* = −0.390, *p* = 0.151, R^2^ = 0.152), while mean HR showed a weaker positive relationship (*β* = 0.347, *p* = 0.205, R^2^ = 0.120). Other HRV predictors, including SDNN (*β* = 0.232, *p* = 0.406, R^2^ = 0.054), NN50 (*β* = 0.175, *p* = 0.533, R^2^ = 0.031), pNN50 (*β* = 0.112, *p* = 0.691, R^2^ = 0.013), and RMSSD (*β* = 0.066, *p* = 0.816, R^2^ = 0.004), showed small or negligible effects. Additional time-domain HRV variables, including the stress index (i.e., the square root of Baevsky’s stress index, as provided by the Kubios software, where higher values reflect cardiovascular stress and greater sympathetic tone) (*β* = 0.026, *p* = 0.926, *R*^2^ = 0.001), TINN (*β* = 0.085, *p* = 0.763, R^2^ = 0.007), Triangular Index (*β* = 0.117, *p* = 0.678, R^2^ = 0.014), and min HR (*β* = 0.100, *p* = 0.724, R^2^ = 0.010), also demonstrated weak, nonsignificant associations. Overall, no single autonomic measure significantly predicted changes in anagram performance.

### 4.2. Divergent

**Figure 1:**
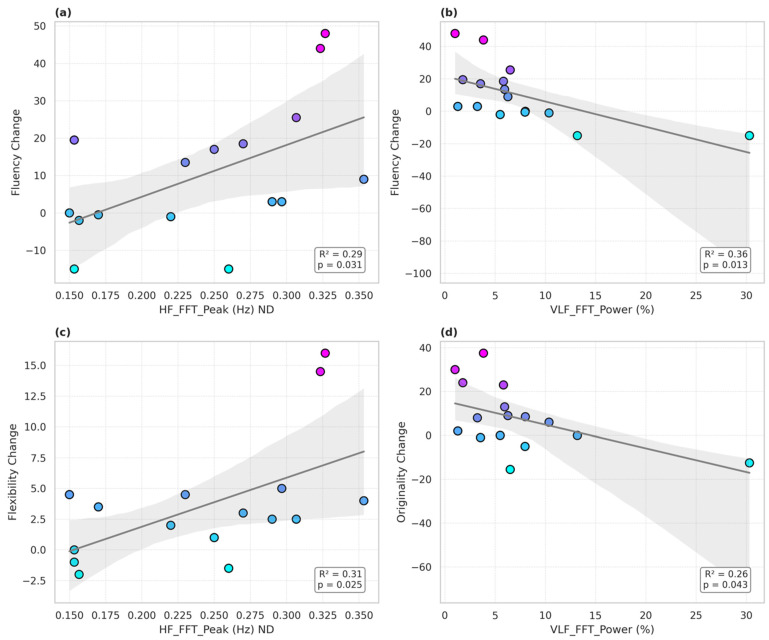
Several HRV frequency-domain indices significantly predicted stimulant-related changes in divergent thinking. Higher baseline HF FFT peak frequency predicted a greater improvement in both fluency (**a**) (*β* = 138.56, *p* = 0.031, *R*^2^ = 0.29) and flexibility (**c**) (*β* = 39.93, *p* = 0.025, *R*^2^ = 0.31) on the TTCT. In contrast, VLF FFT power expressed as a percentage of total power negatively predicted changes in fluency (**b**) (*β* = −1.56, *p* = 0.013, *R*^2^ = 0.36) and originality (**d**) (*β* = −1.08, *p* = 0.043, *R*^2^ = 0.26). These results suggest differential contributions of the parasympathetic and sympathetic components of HRV to stimulant-induced creativity gains in individuals with ADHD.

The hypothesized time-domain HRV variables including pNN50 did not predict psychostimulant-induced changes in performance on the TTCT. Analyses revealed that several baseline frequency-domain HRV indices significantly predicted changes in divergent thinking performance following stimulant administration. Notably, the HF FFT peak frequency was associated with greater stimulant-induced improvements in TTCT fluency (*β* = 138.56, *p* = 0.031 *, *R*^2^ = 0.29) (Figure 1a) and flexibility (*β* = 39.93, *p* = 0.025 *, *R*^2^ = 0.31) (Figure 1b). In contrast, greater VLF FFT power (expressed as a percentage of total spectral power) was negatively associated with changes in fluency (β = −1.56, *p* = 0.013, *R*^2^ = 0.36) (Figure 1c) and originality (*β* = −1.08, *p* = 0.043 *, *R*^2^ = 0.26) (Figure 1d). However, the findings from these secondary measures did not survive the correction of multiple measures. The other findings, though, begin to suggest that distinct branches of autonomic regulation may have differentially influenced stimulant-related gains in creative cognition among individuals with ADHD.

## 5. Discussion

In a previous study, our lab found that stimulant medication enhanced performance on divergent tasks in individuals with ADHD [29]. No effect was seen, however, on performance on convergent tasks. In this follow-up study, the relationship between convergent and divergent creative thinking and how it relates to ANS functioning in people with ADHD taking stimulant medication was examined. Our a priori hypothesis was not confirmed: creativity gains were not significantly predicted by baseline HRV pNN50 for the anagram task which measured convergent creativity; the pNN50 was selected as previous work with adrenergic agents in a patient population had revealed an association with the degree of change on anagram performance [26]. In addition to there being no effect of stimulants seen on performance overall [29], pNN50 was not predictive of the difference in performance between drug conditions. With the nonsignificant findings for HRV and the creativity results, the findings aligned with the dopaminergic contributions to divergent thinking driving the influence of stimulants on divergent creativity performance in ADHD. Our exploratory analysis found that VLF was negatively associated with both fluency and originality, indicating that greater sympathetic activity may interfere with the generation of novel and diverse ideas. VLF-HRV is linked to sympathetic nervous system activation and regulatory processes, suggesting that autonomic dysregulation (i.e., excessive sympathetic influence) might compromise cognitive flexibility and originality in creative tasks [34]. Additionally, the HF fast Fourier transform (FFT) peak frequency was positively associated with both flexibility and fluency scores. HF-HRV reflects respiratory sinus arrhythmia and is a marker of parasympathetic tone. Since the HF FFT peak frequency identifies the dominant oscillatory pattern within this band, its association with improved divergent thinking may reflect a beneficial role of parasympathetic regulation in supporting cognitive flexibility and creative idea generation. However, as these measures were secondary, and the findings did not survive correction for multiple measures, this must be re-examined in future studies before conclusions can be drawn. The overall lack of any of the hypothesized HRV variables being associated with stimulant-related improvements on divergent task performance may lend support to the hypothesis that the effects of stimulants on divergent task performance are DA-related.

Future research is needed to better understand the broader implications of these findings. While divergent creativity may be largely influenced by DA effects, it may still be possible that adrenergic activity impacts both convergent cognitive flexibility and idea generation through one or more pathways (top-down or bottom-up) at greater levels of activation. This aligns with research suggesting that heightened sympathetic activation can restrict divergent idea generation [35].

Overall, enhanced performance on divergent thinking tasks observed in ADHD individuals on stimulant medication may be driven by enhanced dopaminergic activation, facilitating the increased fluency of new ideas. The precise mechanisms underlying divergent thinking in individuals with ADHD remain unclear. To elucidate this uncertainty, it would be of particular interest to utilize neuroimaging techniques to investigate neurotransmitters (NE and DA) in relation to creativity-based tasks or measures. Our results may be of interest to individuals with ADHD and clinicians prescribing stimulant medication who may be concerned with how NE and DA transmission affect creative endeavors. Notably, the present findings provide preliminary evidence suggesting that noradrenergic influences may not play a primary role in modulating the effects of stimulants on creative performance in ADHD. Integrative approaches are needed that account for the multifaceted nature of creative cognition. Future work should explore, in a more systematic manner, how the dopaminergic and noradrenergic systems impact creativity, but the present study begins to suggest that it is the dopaminergic system that is driving the effect of stimulants on creativity in ADHD.

## Figures and Tables

**Table 1:** Participant demographics and medication characteristics. The table summarizes the sample characteristics including age, gender, education, and income distribution for the 16 participants. Medication type and dosage data are provided for individuals prescribed stimulant treatments during the study. Reported dosage values reflect the group means (mg) and standard deviations (SDs) for each medication class.

Demographic Category	Subcategory	Frequency (n)
Age Range	19–42 years (M = 27.9; SD = 6.7)	n = 17
Gender	Female	n = 9
	Male	n = 8
Education Level	Undergraduate	n = 13
	Post-Bachelor’s	n = 1
	Graduate	n = 2
Income per Year	<$25,000	n = 9
	$26,000–$75,000	n = 3
	Undisclosed	n = 4
Medication Type	Amphetamine-based Stimulants	n = 10 (M = 25.5 mg; SD = 6.5 mg)
	Lisdexamfetamine	n = 5 (M = 44 mg; SD = 10.2 mg)
	Methylphenidate-based Stimulants	n= 1 (M = 36 mg; SD = N/A)
	Dexmethylphenidate	n = 1 (M = 23 mg; SD = N/A)

## Data Availability

The data presented in this study are available on request from the corresponding authors due to privacy reasons.
